# Adherence to Dietary and Lifestyle Guidelines Among Women With a History of Gestational Diabetes Mellitus and the Influence of a Student‐Led Dietetic Clinics

**DOI:** 10.1002/fsn3.70076

**Published:** 2025-03-04

**Authors:** Klaudia Illenberger, Julia Sekula, Robyn Lawrence

**Affiliations:** ^1^ Department of Nutrition and Dietetics, Faculty of Medical and Health Sciences The University of Auckland Auckland New Zealand; ^2^ Beef + Lamb New Zealand Inc. Auckland New Zealand; ^3^ School of Sport, Exercise and Nutrition Massey University Auckland New Zealand

**Keywords:** diet, gestational diabetes mellitus, history of gestational diabetes mellitus, lifestyle, physical activity, student‐led dietetic clinics

## Abstract

Women with a history of gestational diabetes mellitus have an increased risk of developing type 2 diabetes. Healthy diet and lifestyle habits may contribute to reducing this risk. This study aimed to describe dietary intake and lifestyle habits of women with a history of gestational diabetes mellitus and determine the impact of nutrition and lifestyle counseling on dietary intake and lifestyle goals. This retrospective cohort study included 32 women with a history of gestational diabetes mellitus 6 months postpartum who attended the student‐led nutrition clinic between 01 June 2021 and 31 August 2022. Dietary intake, lifestyle, and physical activity habits were extracted from student dietitians' notes. The mid‐*p*‐value McNemar's test was used to determine whether attendance at the student‐led dietetic clinic was associated with a change in the proportion of women meeting the guidelines. Fifteen percent (*n* = 5) of women did not meet any food group recommendations prior to any interventions. Most women (46.8%) engaged in less than the recommended level of physical activity and more than a quarter (28.1%) did not engage in any physical activity at their initial consultation. After attending at least one consultation, a greater proportion of women met recommendations for at least one food group (41.1% vs. 18.7%, *p* = 0.256) and a greater proportion of women met physical activity guidelines (60% vs. 25%, *p* = 0.125). In this cohort of postpartum women with a history of gestational diabetes mellitus, adherence to the Ministry of Health Eating and Activity guidelines was poor. Positive changes towards adherence are possible after attendance at a student‐led nutrition and dietetic clinic.

## 1 Introduction

Obesity and diabetes are increasingly affecting people of all demographics throughout the world (World Health Organisation 2023; World Health Organisation 2021). Gestational diabetes mellitus (GDM) has also been increasing both worldwide and within New Zealand (NZ) (Lawrence et al. 2019; McIntyre et al. 2019). GDM is a metabolic condition described as glucose intolerance first diagnosed in pregnancy, with blood glucose levels below the threshold of overt diabetes but above the normal range (American Diabetes Association 2021; Ministry of Health 2014). GDM is the most common metabolic disorder during pregnancy, affecting around 6% of pregnancies in NZ (Lawrence et al. 2019). Worldwide, the prevalence ranges from 1% to more than 30%, depending on ethnic group and diagnostic criteria used (McIntyre et al. 2019). Additional factors contributing to the rise in the prevalence of GDM include a rise in maternal age during pregnancy, obesity rates, dietary intake, and lifestyle habits (Fu and Retnakaran 2022; Sweeting et al. 2022).

GDM usually resolves postnatally (American Diabetes Association 2010); however, it can have long‐term health consequences for both the mother and child (Damm 2009; Fraser and Lawlor 2014). For the mother, GDM increases the risk of developing type 2 diabetes mellitus (T2DM) by sevenfold, with the highest risk being 3–6 years after pregnancy (Song et al. 2018). T2DM is a chronic disease characterized by high blood glucose levels and insulin resistance (Galicia‐Garcia et al. 2020). Long‐term consequences of T2DM, especially if not well controlled, include blindness, kidney failure, and a high incidence rates of cardiovascular events (World Health Organisation 2016).

Due to the increased risk of developing T2DM, the Ministry of Health (MoH) in NZ recommends that women with a history of GDM be offered an HbA1c screening test 3 months postpartum and annually thereafter (Ministry of Health 2014). When evaluating post‐natal screening uptake nationwide, a recent NZ study by Sise et al. found that only 40.9% received an HbA1c test or the Oral Glucose Tolerance Test (OGTT) within 3 months, 53.3% within 6 months, and 61% at 12 months postpartum (Sise et al. 2022). With the rising prevalence of GDM and the poor screening rates of T2DM postpartum in NZ, any potential mitigation of the progression from GDM to T2DM would improve the future health of these women and potentially result in significant cost savings in healthcare (Pricewaterhouse Coopers 2021). There are considerable amounts of international research that have found a positive correlation between a high‐quality dietary intake, physical activity, and reducing the risk of T2DM in women with a history of GDM (Ratner et al. 2008; Retnakaran et al. 2010; Tobias 2018). However, in NZ, no studies have specifically evaluated the impact of dietary and physical activity interventions on reducing the risk of T2DM in women with a history of GDM.

The NZ MoH Eating and Activity Guidelines are the current evidence‐based guidelines on healthy eating and physical activity for NZ adults, pregnant, and breastfeeding women (Ministry of Health 2020). There are similarities in the MoH Eating and Activity Guidelines and the diets associated with T2DM risk reduction in women with a history of GDM (Ministry of Health 2020; Rayner et al. 2020; Sullivan et al. 2012; Tobias et al. 2016). This includes the promotion of fruit, vegetables, whole grains, seafood, lean meats, and healthy fats. Therefore, it is plausible that following the Eating and Activity guidelines may help to reduce the risk of developing T2DM in women with a history of GDM.

In NZ, registered dietitians are qualified healthcare professionals who deliver evidence‐based food and nutrition guidance and provide practical solutions to minimize disease burden (Andersen et al. 2018). Adequate training of student dietitians is essential to maintain and improve the high standards registered dietitians provide. To achieve this, students are often placed in hospital placement and student‐led clinics. Student‐led clinics increase care provision by increasing accessibility and reducing the burden of waitlists to healthcare facilities (Buckley et al. 2014). Students deliver most of the clinical sessions, with a qualified practitioner providing direct or indirect supervision.

This retrospective cohort study describes the postpartum dietary intake and lifestyle habits of women with a history of GDM and evaluates the impact of nutrition and lifestyle counseling in facilitating the achievement of dietary intake and lifestyle recommendations to reduce the risk of progression to T2DM.

## 2 Methods

Participants were recruited from the student‐led Nutrition and Dietetic Clinic between 01 June 2021 and 31 August 2022. Women were eligible for inclusion if they had a history of GDM in their most recent pregnancy with an initial consultation within 6 months after giving birth. Referrals for postnatal GDM advice were primarily received from Sport Auckland but also accepted from General Practitioners and Te Whatu Ora (regional health) localities within the Auckland region.

Prior to being seen in the student‐led clinic, women with a history of GDM referred by Sport Auckland had one‐to‐one zoom consultations with a Healthy Lifestyle Advisor from the Sport Auckland Green Prescription programme, providing physical activity advice, motivation, and goal setting. Through Green Prescription, women also had access to online video classes that contained many low‐impact exercises. Every 4–6 weeks, or more frequently, depending on individuals' needs, women would receive a ‘check‐in’ text to support them with physical activity progress.

Women with a history of GDM were seen by student dietitians and/or supervising NZ registered dietitians at the student‐led Nutrition and Dietetic teaching clinic via telehealth or kanohi ki te kanohi (in‐person) consultations. As part of usual practice within the student‐led clinic, initial consultations were allocated 60‐min time slots, where a student dietitian would use the Nutrition Care Process (Academy of Nutrition and Dietetics 2023) to collect and document the nutritional assessment, including client history, anthropometric measurements, biochemical data, nutrition‐focused physical findings, a food or nutrition‐related history, and nutrition diagnosis. Following the initial consultation, clients were offered a 30‐min follow‐up, if clinically indicated, to check progress and continue to work towards the patient's dietary intake and lifestyle goals. After consultations, the student dietitians documented their assessment and consultation, which were approved by a supervising NZ registered dietitian prior to being uploaded onto the clinic's patient management software.

Eligible women who attended the student‐led clinic were contacted via phone or email by the researcher following their first consultation. Following the contact, women were sent a unique link containing the Participant Information Sheet and the electronic Consent Form via RedCap. If the women agreed to be part of the study but did not sign the consent form, women were sent either a text or phone reminder up to three more times. If the women signed a consent form, a unique identifier was assigned to de‐identify data for analysis.

If women had more than one follow‐up consultation, only their first and last diet history were used in analyses. When categorizing food servings, the MoH Eating and Activity Guidelines examples were used as a guide to count servings consumed by each participant (Ministry of Health 2020). The recommended number of portions for each of the food groups vary according to breastfeeding status and are summarized along with physical activity recommendations in Table S1.

Maternal socio‐demographics, physical activity, and adherence to food groups are reported as the frequency (%) for categorical variables. Ethnicity was categorized according to Statistics NZ (Stats NZ 2018). The mid‐*p*‐value McNemar's test was used to determine whether attendance at the student‐led dietetic clinic was associated with a change in the proportion of women meeting recommendations for the number of serves from each food group and physical activity between the first and final follow‐up consultation. OmniCalculator was used to complete mid‐*p*‐value McNemar's test (Szczepanek 2022). Results are reported using *p*‐values and 95% confidence intervals (CI). A two‐sided *p*‐value of < 0.05 was considered statistically significant. Ethical approval was obtained from the Health Research Ethics Committee (AHREC) on the April 13, 2022, reference number AH23630. This article followed the 2020 PRISMA guidelines version.

## 3 Results

A total of 58 eligible women attended the student‐led Nutrition and Dietetic clinic during the study period. Thirty‐two women consented to take part in this study, 10 declined to participate, and 14 were not able to be contacted. Fifteen (46.9%) women attended one consultation, and 17 (53.1%) attended more than one consultation. Of those who had multiple consultations, there was a median of two follow‐up consultations, with a mean of two and a half months between consultations. The sociodemographic characteristics of women who participated in this study are shown in Table 1. Half (50%, *n* = 28) of the women were aged between 35 and 39, more than a third (39.2%, *n* = 22) were of Indian ethnicity. Over half (55.2%, *n* = 31) of the women were multiparous, and more than half (53.5%, *n* = 30) breastfed their baby at the time of their initial consultation. Almost half (48.2%, *n* = 27) of the women were seen within 3–4 months postpartum.

**TABLE 1:** Characteristics of women with a history of GDM referred to the student‐led nutrition and dietetic clinic^a^.

*n* (%)	Women seen in clinic	Women referred but not seen in clinic
	56 (62.2)	34 (37.7)
Age group (years)		
25–29	4 (7.1)	8 (23.5)
30–34	20 (35.7)	13 (38.2)
35–39	28 (50.0)	11 (32.3)
40 and over	4 (7.1)	2 (5.8)
Self‐prioritized ethnicity		
NZ European	9 (16.0)	4 (11.7)
Māori	0 (0)	1 (2.9)
Pacific peoples	2 (3.5)	7 (20.5)
Asian	9 (16.0)	9 (26.4)
Middle Eastern/Latin American/African	22 (39.2)	9 (26.4)
Other ethnicity	14 (25.0)	4 (11.7)
Health service locality		
Te Toka Tumai Auckland	37 (66.0)	23 (67.6)
Counties Manukau	18 (32.1)	10 (29.4)
Waitematā	1 (1.7)	1 (2.9)
Parity		
One	25 (44.6)	19 (55.8)
Two	27 (48.2)	9 (26.4)
Three	3 (5.3)	4 (11.7)
Four or more	1 (1.7)	2 (5.8)
Type of delivery		
Vaginal birth	19 (33.9)	20 (58.8)
Caesarean	31 (55.3)	13 (38.2)
Feeding type		
Breast feeding	30 (53.5)	17 (50.)
Formula feeding	8 (14.2)	5 (14.7)
Mixed feeding	18 (32.1)	12 (35.2)
Time since birth		
< 1 month	0 (0)	1 (2.9)
1–2 months	18 (32.1)	14 (41.1)
3–4 months	27 (48.2)	12 (35.2)
5 months	5 (8.9)	4 (11.7)
6 months	3 (5.3)	3 (8.8)
8 months	3 (5.3)	0 (0)

*Note:* Missing values have not been included in the column %.

Abbreviation: GDM, gestational diabetes mellitus.

^a^ Data are presented as the number of women (%).

At the first clinic consultation, 3.1% (*n* = 1) of women met recommendations for all five food groups, and 15.6% (*n* = 5) met none (Table 2). After attending at least one consultation, a greater proportion of women met recommendations for at least one food group, from 18.7% (*n* = 6) at the initial consultation to 41.1% (*n* = 7, *p* = 0.256) after one or more consultations. The proportion of women not meeting recommendations for any number of food groups also declined from 15.6% (*n* = 5) to 5.8% (*n* = 1, *p* = 0.317).

**TABLE 2:** Adherence to MoH food group recommendations^a^.

Adherence to food group recommendations	First consultation	Follow‐up consultations	*p* ^b^
	*n* = 32	*n* = 17	
Five food groups	1 (3.1)	0 (0)	0.317
Four food groups	1 (3.1)	0 (0)	0.317
Three food groups	4 (12.5)	2 (11.7)	0.654
Two food groups	15 (46.8)	7 (41.1)	0.738
One food group	6 (18.7)	7 (41.1)	0.256
No food groups	5 (15.6)	1 (5.8)	0.317

*Note:* Data presented as number of women (%).

Abbreviation: MoH, Ministry of Health.

^a^ Ranges are according to the MoH Eating and Activity Guidelines.

^b^ From McNemar's analyses with mid‐*p* binomial test.

Adherence to the MoH Eating and Activity Guidelines for each food group are shown in Table 3. The greatest adherence to recommendations at both initial and follow‐up consultations was to the protein (legumes, nuts, seeds, fish and other seafood, eggs poultry, and the lean meat) food group, where 68.7% (*n* = 22) met recommendations at initial assessment and 82.3% (*n* = 14) met this recommendation at follow‐up consultation. The poorest adherence was seen in the vegetable food group, where 9.3% (*n* = 3) and 0% (*n* = 0) meet recommendations at first and follow‐up consultations, respectively. There was an observed improvement in meeting food recommendations in fruit, milk and milk products, and protein (legumes, nuts, seeds, seafood, eggs, poultry, and lean meat) food groups after attending the student‐led clinic at least once, although this was not statistically significant (Table 3). There were statistically significantly fewer women that met the recommended number of serves for grains following a follow‐up consultation (*p* < 0.001).

**TABLE 3:** Adherence to MoH specific food group recommendations^a^.

Adherence to food group recommendations	First consultation	Follow‐up consultations	*p* ^b^	95% CI
	32 (100.0)	17 (53.1)		
Fruit	7 (21.8)	9 (52.9)	0.125	−49.46, 2.40
Vegetables	3 (9.3)	0 (0.0)	0.5	−5.30, 17.07
Grain foods	20 (62.5)	1 (5.8)	< 0.001	41.99, 87.42
Legumes, nuts, seeds, fish and other seafood, eggs, poultry, and lean meat	22 (68.7)	14 (82.3)	0.125	−35.77, 0.47
Milk and milk products	5 (15.6)	3 (17.6)	0.726	−24.49, 36.26

*Note:* Data presented as number of women (%).

Abbreviations: CI, confidence interval; MoH, Ministry of Health.

^a^ Ranges are according to the MoH Eating and Activity Guidelines.

^b^ From McNemar's analyses with mid‐*p* binomial test.

Prior to attending the clinic, 46.8% (*n* = 15) of women engaged in less than the recommended level of physical activity (the MoH recommends adults engage in at least 75 min of vigorous or 150 min of moderate physical activity per week (Ministry of Health 2020)) and 28.1% (*n* = 9) of women did not do any physical activity. When comparing physical activity levels at first and final follow‐up consultations, there was an increase in women who met physical activity guidelines after attending the student‐led clinic from 25% (*n* = 32) to 60% (*n* = 10), but this was not statistically significant (Table 4). The most common physical activity was walking (30%, *n* = 7), followed by Green Prescription exercises (17%, *n* = 4) or the combination of both (47%, *n* = 11). Most Green Prescription exercises consisted of low‐impact online classes ranging from 15 to 45 min.

**TABLE 4:** Adherence to MoH physical activity recommendations^a^.

Physical activity engagement	First consultation	Follow‐up consultations	*p* ^b^	95% CI
	*n* = 32	*n* = 10		
No physical activity	9 (28.1)	1 (10.0)		
Below recommendation	15 (46.8)	3 (30.0)		
Met recommendation	8 (25.0)	6 (60.0)	0.125	58.40, −1.60

*Note:* Data presented as number of women (%) for available data.

Abbreviations: CI, confidence interval; MoH, Ministry of Health.

^a^ The MoH recommends adults engage in at least 75 min of vigorous or 150 min of moderate physical activity per week.

^b^ From McNemar's analyses with mid‐*p* binomial test.

## 4 Discussion

Women with a history of GDM are at a substantial risk of developing T2DM postpartum, and there are currently limited healthcare resources directed toward preventing T2DM for these women in NZ (Song et al. 2018). Extensive research has shown the relationship between dietary intake, lifestyle habits, and reducing the risk of T2DM (Bao et al. 2016; D'Arcy et al. 2020; Tobias et al. 2012).

In this study, women with a history of GDM had poor adherence to the MoH Eating and Activity guidelines (Ministry of Health 2020) in the postpartum period. Fewer than 4% of women met all the food group recommendations within 6 months postpartum. This is similar to the findings of other research during pregnancy, where < 4% of NZ women with GDM adhered to all food and nutrition guidelines (Lawrence et al. 2022). This study also evaluated the influence of attending a student‐led dietetic clinic on adherence to the food group recommendations. After attending at least one consultation at the clinic, more participants met recommendations for at least one food group, and the proportion of women not meeting recommendations for any number of food groups decreased. The student‐led nutrition and dietetic clinic has the potential to lead to positive changes toward improving the dietary intake of women with a history of GDM.

In over half the participant group, the greatest adherence to recommendations seen postpartum was towards the protein (legumes, nuts, seeds, seafood, eggs, poultry, lean meat intake) and grain food groups. In contrast, adherence was low for the fruit, vegetable, and milk and milk product food groups. When compared to women with GDM during pregnancy in NZ, adherence to the fruit, and milk and milk product groups were the highest, and adherence to the vegetable, grain, and protein (legumes, nuts, seeds, seafood, eggs, poultry, and lean meat) groups were the lowest (Lawrence et al. 2022). During the initial clinical consultation, more than half of the women in our study met the food group recommendation for intake of protein (legumes, nuts, seeds, seafood, eggs, poultry, and lean meat). After at least one consultation at the student‐led clinic, there was an observed increase in adherence, where more than three‐quarters of the women met recommendations for this food group, although this was not statistically significant. In a study including 184 women with a history of GDM, Hwang et al. found women living with diabetes had a higher consumption of protein than women with normoglycemia postpartum (Hwang et al. 2011). Some protein foods, such as animal protein or processed meats, have been associated with an increased risk of developing T2DM (Tian et al. 2017). Whereas plant‐based foods and reduced fat milk and milk products have been found to be protective against T2DM (McMacken and Shah 2017; Alvarez‐Bueno et al. 2019). However, data collected during this study did not differentiate between plant and animal‐based protein foods consumed; therefore, it is unclear how this finding might impact the risk of developing T2DM.

During the initial consultation, more than half of the women adhered to the grain food group recommendations. Interestingly, during pregnancy, only a quarter of women met their recommendations for this food group (Lawrence et al. 2022). This difference could be explained by women being more restrictive than they should be after receiving a GDM diagnosis. Whereas our findings suggest that perhaps women allowed themselves to consume carbohydrate foods again during the postpartum period (Teh et al. 2021). In contrast, the cross‐sectional study by Morrison et al. analyzed diets of women with a history of GDM and found low adherence to grain foods (Morrison et al. 2012). After attending the student‐led clinic, significantly fewer women met their requirements and “under consumed” the grain food group following a consultation.

The intake of milk and milk products in our study were below the recommended number of serves for women postpartum. A cross‐sectional observational study in NZ by Brown et al. included 458 women through pregnancy and the first 6 months postpartum found dairy and dairy product consumption reduced during lactation compared to during pregnancy (Brown et al. 2020). Women reportedly reduced their intake due to fear of causing the infant discomfort or to minimize allergic reactions in their babies. In contrast, a NZ cohort by Lawrence et al. found that dairy and dairy product consumption was the second highest food group met, with half of the women with GDM during pregnancy meeting the recommendations (Lawrence et al. 2022). These findings may suggest that perhaps women change their nutritional habits postpartum. Moreover, low or complete avoidance of milk and milk products could cause deficiencies, which could lead to serious health consequences for the mother and the baby. Women may also have limited awareness about evidence‐based practices, recommendations, and babies' health (Brown et al. 2020).

Similar to milk and milk products, adherence to vegetable recommendations was low, with only 9.3% of women meeting their requirements. Similar international research is also consistent with our findings (Zehle et al. 2008; Kieffer et al. 2006), suggesting that while pregnant women may understand the importance of vegetable consumption and make efforts to improve their dietary intake, there may be a lack of knowledge and skills among postpartum women regarding recommended quantities and cooking techniques for vegetables they enjoy (Zehle et al. 2008).

Alongside knowledge and skills, environmental factors such as the cost of living, food insecurity, income, and low socioeconomic environments can also influence dietary choices and behaviors (Stone et al. 2024; Hill and Peters 1998).

Physical activity has numerous health benefits, with an opportunity to improve quality of life, but also to prevent T2DM long‐term (Ministry of Health 2020; Sahrakorpi et al. 2022; Bassuk and Manson 2005). Most women participated in some physical activity; however, only a quarter met the MoH Eating and Activity Guidelines recommendations of two and a half hours of moderate or an hour and a quarter of vigorous physical activity throughout the week (Ministry of Health 2020). More than a quarter of women did not engage in any physical activity postpartum.

There was an observed increase, where more than half of the women met the physical activity guidelines after attending the student‐led clinic, although not statistically significant. Although the frequency of input from Sport Auckland was not measured in this study, this outcome may be due to a combined effect of seeing a dietitian during pregnancy (Mustafa et al. 2021), receiving frequent support and education in the postpartum period from the student‐led dietetic clinic and the Green Prescription programme run by Sport Auckland. Retnakaran et al. concluded that women with a history of GDM were more likely to adopt change postpartum, which our study also observed (Retnakaran et al. 2010). Women in our study demonstrated great adherence to physical activity after attending at least one consultation in the student‐led dietetic clinic and taking part in the educational sessions from the Green Prescription programme.

The main strength of this study is that it is the first, to our knowledge, in NZ to explore the dietary and lifestyle behaviors of women with a history of GDM 6 months postpartum. Our study also evaluated the impact of a student‐led dietetic clinic on dietary and lifestyle behaviors. This study took place in the student‐led clinic, which has the potential to reduce the cost barrier for women with a history of GDM and provide telehealth consultations to meet the busy schedules of a mother. This study also observed women within the usual context of the student‐led clinic; therefore, it reflects current practice. Furthermore, the women in our study were contacted after their first consultation to participate in the study, so their dietary and lifestyle responses could exclude potential research confirmation bias.

The study was limited by its small sample size. The clinics are limited by being open only throughout the semester periods, which may affect accessibility. Many women were uncontactable at the point of referral for both the student‐led clinic and Sport Auckland, which limited the sample size of this research. There could be a number of reasons for this difficulty in engaging with this population group. Women might have been uninformed about the referral reasons, lacked awareness of the potential risk of T2DM, or experienced fear concerning the potential diagnosis of T2DM. It could also be that the contact is too soon, and women may prioritize the care of the baby over their own health.

It is probable that our study is impacted by sampling bias, as the women who took part in our study from the student‐led clinic displayed a proactive approach toward their health by actively responding and consenting to participate, thereby increasing the likelihood of making changes. Māori and Pacific Island ethnicities were especially underrepresented in this study. However, our participant demographic is similar to the Te Toka Tumai Auckland demographic profile, where referrals predominantly originated Some women may have also had prior perceptions of student‐led clinics, such as only wanting to receive health care advice from a registered dietitian rather than predominantly from a student or not knowing the concept of a student‐led clinic.

Our study also did not evaluate overconsumption, which would demonstrate further risks for NZ women with a history of GDM.

This study shows that women with a history of GDM in NZ overall have poor adherence to the MoH Eating and Activity guidelines within 6 months postpartum, similar to other studies exploring these behaviors (Morrison et al. 2012; Evans et al. 2010; Fehler et al. 2007; Jusoh and Tengku Ismail 2022; Lee et al. 2020). This has significant implications for their future risk of T2DM. Attending the student‐led clinic improved adherence to the MoH Eating and Activity Guidelines and physical activity levels in conjunction with input from the team at Sport Auckland. Without the appropriate support of nutritional and lifestyle interventions, women with a history of GDM could be on a direct trajectory for developing T2DM. Future studies should investigate in more depth the dietary intake and lifestyle habits of women with a history of GDM to further evaluate the risk of developing T2DM and understand how to improve healthcare for these women postpartum. Moreover, with this information, optimizing support systems postpartum such as increasing T2DM screening, providing nutritional and lifestyle education, and maternal support within healthcare could reduce the risk factors associated with T2DM in women with a history of GDM.

## Author Contributions

**Klaudia Illenberger:** data curation (equal), formal analysis (equal), investigation (lead), methodology (equal), validation (equal), visualization (lead), writing – original draft (lead), writing – review and editing (equal). **Julia Sekula:** conceptualization (equal), data curation (lead), methodology (equal), project administration (lead), resources (lead), supervision (equal), validation (equal), writing – review and editing (equal). **Robyn Lawrence:** conceptualization (equal), formal analysis (lead), methodology (equal), supervision (equal), validation (equal), writing – review and editing (equal).

## Ethics Statement

This study was obtained from the University of Auckland Health Research Ethics Committee (AHREC) on the April 13, 2022, reference number AH23630.

## Conflicts of Interest

Julia Sekula was employed at the University of Auckland at the time the research was conducted but moved to Beef + Lamb Inc. at the time the manuscript was written. There are no further conflicts of interest to be declared by other authors.

## Supporting information


Table S1.

## Data Availability

The data that support the findings of this study are available from the corresponding author upon reasonable request.
