# Supplementary material for: Adherence to Dietary and Lifestyle Guidelines Among Women With a History of Gestational Diabetes Mellitus and the Influence of a Student‐Led Dietetic Clinics
Source: Food Sci Nutr. 2025 Mar 4;13(3):e70076. doi: 10.1002/fsn3.70076 (PMC11879896; doi:10.1002/fsn3.70076)
Supplement: Supplementary file 1 — Table S1. [file FSN3-13-e70076-s001.docx]

S1. Recommended number of servings per day from each of the food groups for adults from MoH Eating and Activity Guidelines^a^

|  | Vegetables | Fruit | Grain Foods | Legumes, nuts, seeds, fish, other seafood, eggs, poultry or red meat with fat removed | Milk and milk products | Approximate number of additional servings from the five food groups^b^ |
| --- | --- | --- | --- | --- | --- | --- |
| Women | 5 | 2 | 6 | 2.5 | 2.5 | 0-2.5 |
| Lactating | 7.5 | 2 | 9 | 2.5 | 2.5 | 0-2.5 |

^a^Includes an allowance for unsaturated spreads or oils, nuts or seeds (2 servings [14–20g] per day for women). The MoH recommends adults engage in at least 75 minutes of vigorous or 150 minutes of moderate physical activity per week.^20^; ^b^Additional servings may be needed for taller or more active women.
